# Fouling Mitigation of PVDF Membrane Induced by Sodium Dodecyl Sulfate (SDS)-TiO_2_ Micelles

**DOI:** 10.3390/membranes15110330

**Published:** 2025-10-30

**Authors:** Jie Zhang, Shiying Bo, Chunhua Wang, Zicong Jian, Yuehuan Chu, Si Qiu, Hongyan Chen, Qiancheng Xiong, Xiaofang Yang, Zicheng Xiao, Guocong Liu

**Affiliations:** 1 School of Chemistry and Materials Engineering, Huizhou University, 46 Yanda Road, Huizhou 516007, China; anglar@126.com (C.W.); jackyken1984@gmail.com (Z.J.); chuyh26@hzu.edu.cn (Y.C.); qiusi250@gmail.com (S.Q.); chenhy@hzu.edu.cn (H.C.); xiong@hzu.edu.cn (Q.X.); yangxf1989@163.com (X.Y.); 2 College of Life and Environmental Science, Guilin University of Electronic Technology, 1 Jinji Road, Guilin 541004, China; 3 School of Environmental Science and Engineering, South University of Science and Technology of China, No. 1088 Xueyuan Avenue, Shenzhen 518055, China; 4 State Key Laboratory of Pollution Control and Resource Reuse, School of Environmental Science and Engineering, Tongji University, 1239 Siping Road, Shanghai 200092, China; bsytju@tongji.edu.cn

**Keywords:** sodium dodecyl sulfonate, SDS-TiO_2_ micelles, composite membrane, antifouling performance, wastewater treatment

## Abstract

As a favorable hydrophilic additive for antifouling modification of polyvinylidene fluoride (PVDF) membrane, titanium dioxide (TiO_2_) nanoparticles have been applied for years. Sodium dodecyl sulfonate (SDS), a representative anionic surfactant, has been proven to benefit the dispersion of nano-TiO_2_ via an electro-spatial stabilizing mechanism. In this study, various proportionally SDS-functionalized TiO_2_ nanoparticles were adopted to modify PVDF membrane. Dispersion and stability of SDS-functionalized TiO_2_ nanoparticles in casting solutions were evaluated by multiple light scattering technology. The properties and antifouling performance of PVDF/SDS-TiO_2_ composite membranes were assessed. The uniformity of surface pores as well as structures on cross-section morphologies was modified. The finger-like structure of PVDF/SDS-TiO_2_ composite membrane was adequately developed at the SDS/TiO_2_ mass ratio of 1:1. The improved antifouling performance was corroborated by the increasing free energy of cohesion and adhesion as well as the interaction energy barrier between membrane surfaces and approaching foulants assessed by classic extended Derjaguin–Landau–Verwey–Overbeek (XDLVO) theory, the low flux decline during bovine serum albumin (BSA) solution filtration process, and the high critical flux (38 L/(m^2^·h·kPa)) in membrane bioreactor. This study exploits a promising way to modify PVDF membrane applicable to the wastewater treatment field.

## 1. Introduction

In recent years, membrane bioreactors (MBRs) have become a well-regarded technology owing to their high solid–liquid separation efficiency, high-quality effluent and low sludge yield [1]. Ultrafiltration (UF) membranes are commonly used in MBR applications. However, the broader adoption of MBR technology is notably challenged by membrane fouling, which impacts the performance and stability of the operation system in wastewater treatment plants (WWTPs) [2]. The membrane antifouling performance is reported primarily relying on the hydrophilicity and surface charge of membranes [3,4]. Developing antifouling membranes with hydrophilic or negatively charged groups is a fundamental strategy to minimize the fouling puzzle [5,6]. Methods such as grafting [7], coating [8] and plasma treatments [9] are commonly used to enhance surface hydrophilicity and surface charge. Among them, fabrication of composite membranes by introducing inorganic nanoparticles is a convenient strategy to modify membrane surface properties [10]. Introducing hydrophilic additives or inorganic nanoparticles in the preparation process of dope solutions is reported as a straightforward and repeatable operation for the preparation of modified membranes [11].

Titanium oxide (TiO_2_) as an environmentally friendly and chemically stable inorganic nanomaterial has attracted much attention in membrane antifouling modification [12]. Its high water affinity also benefits its wide application in membrane antifouling modification [13]. However, aggregation and sedimentation resulting from the high surface energy might miserably impede antifouling modification of the membrane. Physically blending nanoparticles with a moderate surfactant has been proven to be a valid method to ease the aggregation of TiO_2_ nanoparticles and thus enhance the antifouling performance of membranes in our previous study [14]. SDS, a negatively charged anionic surfactant with a hydrophobic tail and hydrophilic head group, might be an ideal dispersing agent of nanoparticles that can simultaneously modify the hydrophilicity and surface charge of the membrane surface [15]. However, the influence of the SDS content on the stability of the casting solution is still in the blackbox, and the relationship between the dispersion of TiO_2_ nanoparticles with various content of SDS and the resulting membrane antifouling capability has not been addressed clearly yet.

In this study, various mass ratios of SDS-TiO_2_ micelles were prepared by physically blending certain content of TiO_2_ nanoparticles with various dosages of SDS. The existing state of SDS-TiO_2_ micelles in the polymeric homogenous casting solution and original organic solvent was monitored by real-time determined multiple light scattering spectroscopy (MLiSSP) and transmission electron microscopy (TEM), respectively. Morphologies and components of PVDF/SDS-TiO_2_ composite membranes were determined by employing a field emission scanning electron microscope (SEM), energy dispersive X-ray (EDX) spectroscopy and X-ray photo-electron spectroscopy (XPS). The influence of various SDS-TiO_2_ micelles on the permeabilities, hydrophilicities and mechanical properties of PVDF/SDS-TiO_2_ composite membranes were also investigated. Classic XDLVO theory analysis and filtrating process with BSA as the model foulants were conducted to probe into the modification efficiency of antifouling performance. A submerged membrane bioreactor (MBR) under steady operation was utilized to evaluate the antifouling capability for membranes in the actual application of the wastewater treatment process. Critical flux as a guidance indicator of running flux in MBR operation was investigated in this study.

## 2. Materials and Methods

### 2.1. Materials and Experimental Set-Up

Commercial-grade PVDF (Solef^®^ 6020, Mw = 670–700 kDa), used as the polymer material, was purchased from Solvay Corporation (Brussels, Belgium). Dimethylacetamide (DMAC) and dimethylsulfoxide (DMSO), used as solvents, were purchased from Sinopharm (Shanghai, China). Polyethylene glycol (PEG 400) (Sinopharm, Shanghai, China) was used as a pore-forming additive during the membrane fabrication process. Anatase TiO_2_ nanoparticles (about 21 nm), BSA (67 kDa) and sodium dodecyl sulfate (SDS), used as the dispersing surfactant of nanoparticles, were obtained from Sigma-Aldrich (St. Louis, MO, USA). An amount of 1 g/L BSA solution at pH 7.0 adjusted by 0.1 M NaOH or 0.1 M HCl was used as a model foulant in this study. A pilot-scale submerged anoxic/oxic membrane bioreactor (A/O MBR) located in the Quyang municipal wastewater treatment plant (WWTP) of Shanghai and fed with stable raw municipal wastewater was used to evaluate the critical flux of membranes. The schematic diagram was shown in our previous work [16]. The effective volumes of anoxic and oxic zones were 0.41 m^3^ and 0.49 m^3^, respectively. The corresponding hydraulic retention times (HRTs) were 3.6 h and 4.4 h. The sludge retention time (SRT) of 60 d was maintained by daily discharging waste activated sludge. The mixed liquor suspended solids (MLSS) concentration was 6 g/L, and the specific aeration demand (SAD_m_) was set as 1.0 m^3^/(m^2^·h).

### 2.2. Membrane Preparation

PVDF/SDS-TiO_2_ composite membranes with TiO_2_ nanoparticle concentration of 0.15 wt.% were prepared by phase inversion via the immersion precipitation method. The dispersion of TiO_2_ nanoparticles was addressed by introducing various content of anionic surfactant SDS. Mass ratios of SDS/TiO_2_ were set as 0, 0.5, 1 and 2, respectively, and the corresponding PVDF/SDS-TiO_2_ composite membranes were termed as TS1, TS2, TS3 and TS4. Table 1 shows the composition of membranes.

Partial solvent (DMSO and DMAc) was used to dissolve PVDF at 80 °C for 4 days, during which homogenous polymeric solutions were prepared. TiO_2_ nanoparticles were dispersed differently under the electro-spatial stabilizing interaction of SDS in the remaining solvents (employed as a disperse medium). The suspensions of TiO_2_ nanoparticles were stirred ultrasonically at 20 °C for 20 min. Afterward, the suspensions were mixed with the prepared homogenous polymeric solutions at 80 °C for 3 days. Casting solutions of PVDF/SDS-TiO_2_ composite membranes TS1–TS4 were finally prepared hereto. PVDF/SDS-TiO_2_ composite membranes TS1–TS4 were respectively prepared by casting the final solutions onto glass plates and porous fabrics with a casting knife gap of 250 μm and speed of 1.8 m/min. These casting films were then submerged into a coagulation bath (deionized water) within 30 s at room temperature (about 25 °C).

### 2.3. Casting Solution Stability

The multiple light scattering spectroscopy (Turbiscan Tower, Formulaction, Toulouse, France) with a near-infrared light source (λ = 880 nm) was used to investigate the stability of casting solutions at a temperature of 80 °C in this study. The movement of particles within casting solutions was monitored by two synchronous detectors: transmission (*T*) at an angle of 0°, and backscattering (*BS*) at an angle of 135° [17]. The detection monitors scans along the height of the cylindrical glass within the samples from the bottom (0 mm) to the top (about 43 mm) [18]. Backscattering light signal (∆*BS*) and transmission (∆*T*) were analyzed for 4 h with the original signals at 0 s as a reference. ∆*BS* was emphatically considered when ∆*T* was over 0.2%. Turbiscan curve at a height of <3 mm of the glass container shows the sediment formed at the bottom, and the peak thickness indicates the amount of sediment [19]. The Turbiscan Stability Index (TSI) as a statistical quantity was employed to estimate system stability. The higher the TSI value, the lower the stability of the casting solution systems [20]. The TSI value could be calculated by the following equation by taking into account all processing that occurred in the systems [21].

(1) $$TSI=\sqrt{\frac{\sum_{i=1}^{n}{\left(x_{i}-x_{BS}\right)}^{2}}{n-1}}$$

where *x_i_* means the average backscattering for each minute of measurements, *x*_BS_ means average *x_i_*, and *n* is the number of scans.

### 2.4. Membrane Characterization

The dispersion of TiO_2_ nanoparticles under various mass ratios of SDS/TiO_2_ was observed by transmission electron microscopy (TEM, JEM 2011, JEOL Ltd., Tokyo, Japan). The surface and cross-section morphologies of PVDF/SDS-TiO_2_ composite membranes TS1–TS4 were observed by SEM (FESEM, Hitachi S4800, Tokyo, Japan). Surface pore sizes and the distribution as well as surface porosities of PVDF/SDS-TiO_2_ composite membranes were obtained by utilizing Image-pro plus 6.0 software (Media Cybernetics, Rockville, MD, USA). Elemental compositions and the dispersion of SDS-TiO_2_ on the surface of membranes were observed by employing EDX (FESEM, Hitachi S4800, Tokyo, Japan) under 20.0 kV and XPS (Escalab 250 Xi, Thermo, Waltham, MA, USA) with a resolution of 0.68 eV/(C1s). The spectra determined by XPS were calibrated using C1s = 284.6 eV as a reference.

Surface hydrophilicity of each membrane characterized by contact angle was observed by using an optical contact angle measurement system (OCA 15 Plus, Data physics GmbH, Stuttgart, Germany). Zeta potential of each membrane was measured by a streaming potential analyzer (EKA 1.00, Anton-Paar, Graz, Austria). The average size and Zeta potential of the model foulant (BSA in this study) were measured by a Zetasizer analyzer (Nano-ZS90, Malvern Instruments, Malvern, UK). Mechanical properties of PVDF/SDS-TiO_2_ composite membranes were tested by a microcomputer-controlled electric universal testing machine (Sans Material Testing Corporation, Shenzhen, China) at room temperature. The water permeability of each membrane was tested three times under pressure of 0.03 MPa. Porosity was calculated by employing Equation (2).

(2) $$\varepsilon =\frac{m_{1}-m_{2}}{\rho_{w}\cdot A\cdot l}$$

where *m*_1_ and *m*_2_ refer to the weights of the wet and dry membranes (g), respectively. ρ_w_ means the water density (1 g/cm^3^), and *A* means the effective area of the membrane (cm^2^). The membrane thickness *l* (cm) was determined by a micrometer caliper, and each value was measured three times.

Three-dimensional morphologies of membrane surfaces were characterized by an atomic force microscope (AFM) (Dimension 5000, Bruker AXS, Santa Barbara, CA, USA). The AFM micrographs (5 × 5 µm^2^) were captured at room temperature in tapping mode using a dimension icon silicon tip with a force constant of 0.4 N/m and 70 kHz resonance frequency. The surface roughness of each membrane sample was determined as average roughness (Ra), root-mean-square roughness (Rq) and maximum roughness (Rmax).

### 2.5. Antifouling Performance Evaluation

The filtration experiment was carried out by employing a dead-end filtration cell (MSC300, Mosu Corporation, Shanghai, China) at room temperature. BSA solution was filtrated under a vigorous stirring rate of 500 rpm. The pure water flux of fouled membrane after the process of physical washing was determined under the pressure of 0.03 MPa. Flux recovery can be obtained by comparison to the initial permeability.

As reported in our previous study, the adsorption of foulants on solid surfaces of PVDF/SDS-TiO_2_ composite membranes can be evaluated by XDLVO theory [22]. DI water, formamide and diiodomethane, with known surface tension parameters, were utilized as three probe liquids. Contact angles of membrane surfaces using the three probe liquids were determined. The electron acceptor parameter γ^+^ and the electron donor parameter γ^−^, as well as the calculated surface tension parameters (γ^TOT^, the total tension parameter) and the components (γ^AB^, the AB component and γ^LW^, the LW component) of membranes, can be obtained by applying the corresponding contact angles and surface tension parameters of the probe liquids. The total free energy of cohesion per unit area of membranes (ΔG^TOT^), along with the free energy of adhesion between membranes and foulants, can be used to assess the antifouling capacity of PVDF/SDS-TiO_2_ composite membranes. The interaction energy (U) between membrane surfaces and the approaching foulants provided a theoretically quantitative insight into antifouling performance during the fouling process.

The critical fluxes of PVDF/SDS-TiO_2_ composite membranes were determined in a similar device, which was located at Quyang Municipal Wastewater Treatment Plant (WWTP) of Shanghai. Step duration and incremental flux were set as 15 min and 3 L/(m^2^·h), separately. The initial flux was set as 27 L/(m^2^·h). According to the step-wise method, critical flux was defined as the flux above which the increase in TMP exceeded 0.4 kPa in one step duration (15 min). The critical flux of each membrane was measured three times at approximately 20 °C. The physicochemical characteristics of the influent wastewater for critical flux measurement is displayed in Table S1.

## 3. Results and Discussion

### 3.1. Casting Solution Stability Analysis

**Figure 1:**
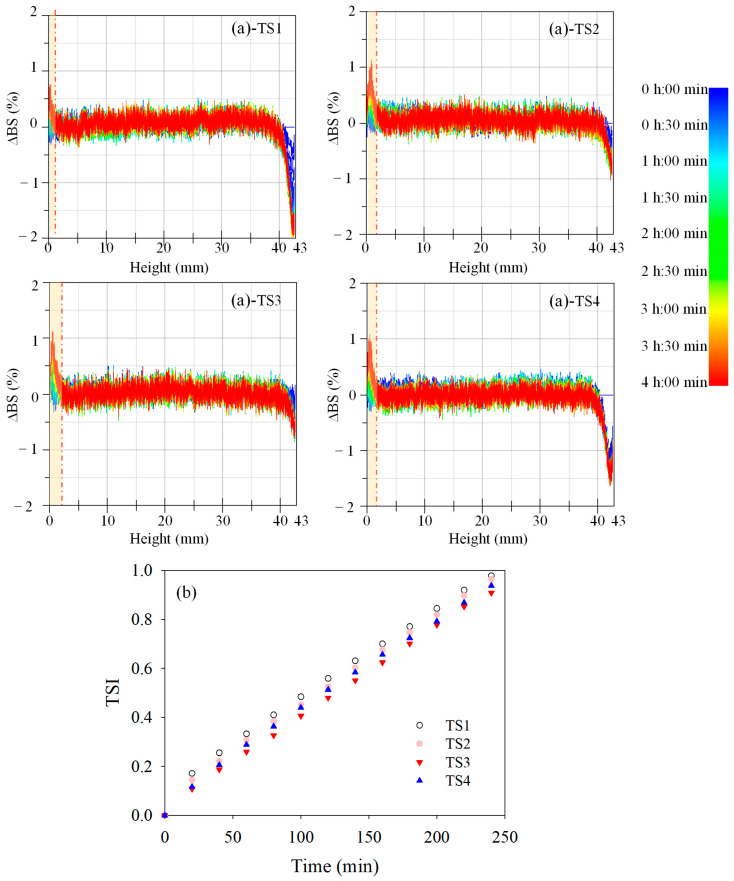
(**a**) The backscattering intensity profiles along the sample height, and (**b**) TSI of casting solutions of membranes TS1–TS4 during the measurement period of 4 h.

The role of SDS as a dispersing agent capable of reducing the nanoparticles’ agglomeration/aggregation in casting solution was monitored by a Turbiscan device in this study. The backscatter curves of the multiple light scattering spectroscopy reveal the phenomenon of agglomeration, flocculation and sedimentation of nanoparticles in the casting solutions. The presence of the backscattering peaks at a low height (shown in Figure 1a) suggests the sedimentation process of nanoparticles, and the peaks at a high height indicate the clarification of casting solutions at the top [23]. The higher strength of the peaks at the low height and the high height indicate the more severe sedimentation of nanoparticles and the obvious clarification of the casting solutions, both implying the lower stability of the casting solution systems. The peak at the low height of TS1 and TS4 might be attributed to the agglomeration of bare nanoparticles and the compression of SDS-TiO_2_ micelles. The highest peak strength at the high height of TS1 indicated the lowest stability of casting solution systems, which was followed by TS4. However, the lowest peak strength at the high height of TS3 showed the highest stability. The results were in accordance with the TSI values (shown in Figure 1b). The highest TSI values throughout the whole determination for sample TS1 revealed the lowest stability. However, the lowest TSI values for sample TS3 showed the adverse stability result, which testified to the excellent stabilization of SDS at the mass ratio of SDS/TiO_2_ of 1:1.

### 3.2. Membrane Characterizations

**Figure 2:**
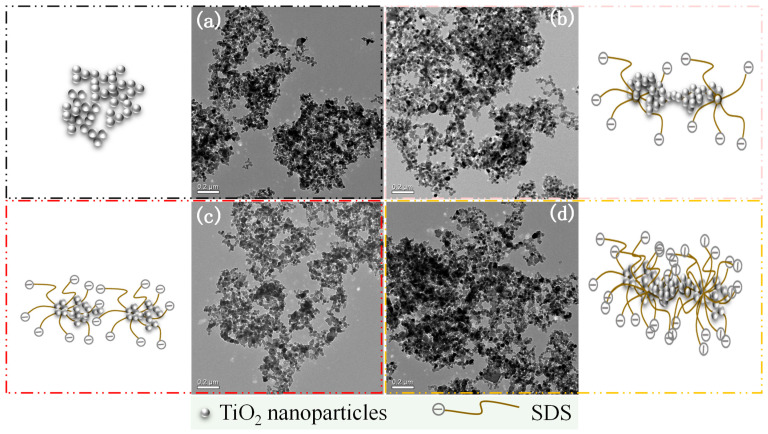
Scheme and the dispersion of TiO_2_ under the interaction of SDS for PVDF/SDS-TiO_2_ composite membranes TS1–TS4 (**a**–**d**).

Figure 2 exhibits the dispersion of TiO_2_ nanoparticles as well as the schematic mechanism in the disperse systems. The surfactant SDS could be adsorbed onto the surface of TiO_2_ nanoparticles, forming the micelles with central (TiO_2_ nanoparticles)-shell (surfactant, SDS) structure, which might form a “separative layer” around the NPs and thus mitigate the agglomeration of the central NPs via steric/electrostatic repulsions interaction [24,25]. Compared to the disperse system without SDS for PVDF/SDS-TiO_2_ composite membrane TS1, the agglomeration of TiO_2_ nanoparticles in the disperse systems for membranes TS2 and TS3 enhanced gradually by the act of the electrostatic or steric repulsions between the surfactants adsorbed on the nanoparticle surfaces. With the mass ratio of SDS/TiO_2_ of 1, the micelles of nanoparticles and SDS might have formed evenly, manifesting the preferred dispersion. With the increase in SDS for PVDF/SDS-TiO_2_ composite membrane TS4, the system showed the severe agglomeration of TiO_2_ nanoparticles via the entanglement of surfactants. The dispersion results detected by TEM also illustrated the fact that the adsorption of surfactants to NPs and micelle formation were concentration-dependent. The obvious agglomeration of bare TiO_2_ nanoparticles for TS1 and superfluous SDS for TS4 as well as the preferably dispersed SDS-TiO_2_ micelles for TS3 shown in Figure 2 were also verified by the results displayed in Figure 1a. The results determined by Turbiscan and TEM both manifested the fact that the mass ratio of SDS/TiO_2_ played an important role in the dispersion of TiO_2_ nanoparticles.

**Figure 3:**
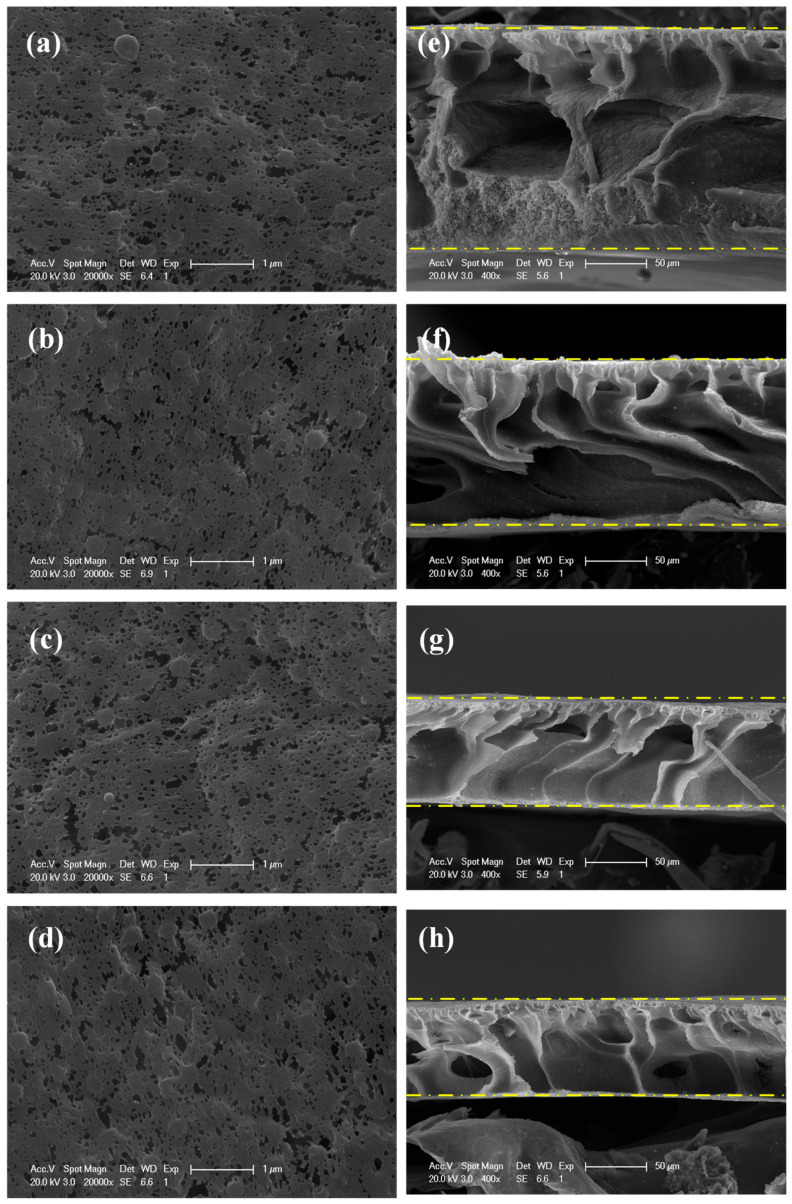
Surface morphologies (**a**–**d**) and cross-section morphologies (**e**–**h**) of PVDF/SDS-TiO_2_ composite membranes TS1–TS4.

Surface morphologies of membranes are shown in Figure 3a–d. Surface porosities and pore sizes observed from the surface morphologies are shown in Figure S1a and Figure S1b–e, respectively. Surface morphologies of PVDF/SDS-TiO_2_ composite membranes TS1–TS4 displayed no significant difference and the surface average pore sizes fluctuated between 0.058 and 0.063 μm. Membrane TS3 exhibited a relatively high surface porosity, which might be owing to the balanced structure of micelles. The decreased surface porosity of membrane TS4 might be attributed to the fact that the overdosage SDS adsorbed on the surface probably entangled and cross-linked with the PVDF polymers and thus blocked pores on the surface.

**Figure 4:**
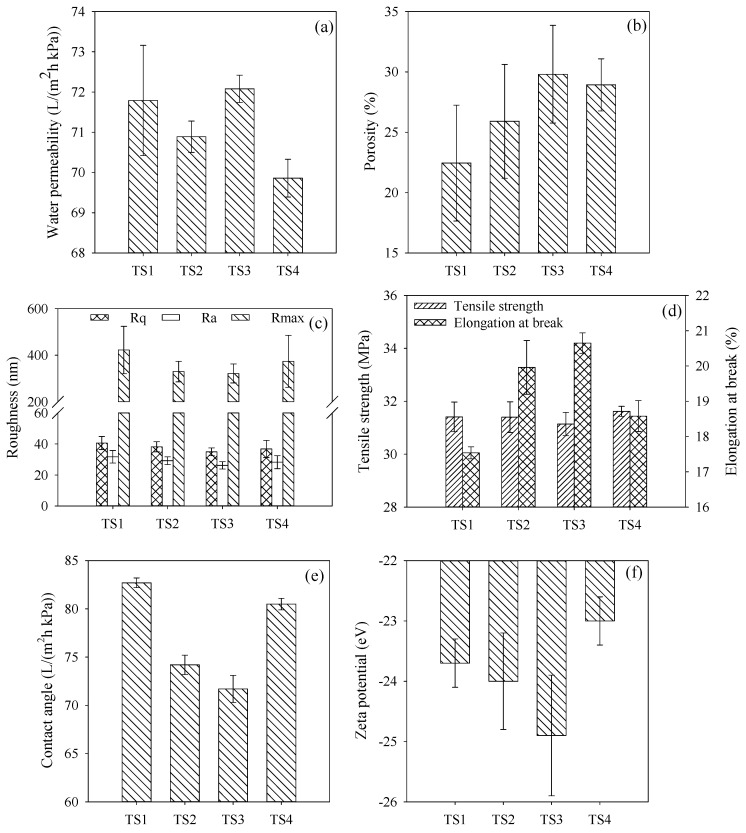
Properties of membranes TS1–TS4 (*n* = 3): (**a**) water permeabilities, (**b**) cubical porosities, (**c**) roughness, (**d**) mechanical strength, (**e**) contact angle and (**f**) Zeta potential.

Figure 3e–h—displays the cross-section morphologies of PVDF/SDS-TiO_2_ composite membrane TS1–TS4. Membrane TS1 exhibited a relatively spongy structure at the bottom, which might be attributed to a slow exchange velocity between solvents and coagulating bath during the phase inversion process. The addition of a small amount of SDS in the casting solution resulted in considerably thinner top-layer and more porous sub-layer membranes in comparison to PVDF/TiO_2_ composite membrane TS1. The SDS-TiO_2_ micelles with the hydrophilic tail might accelerate the exchange velocity in the hydrophilic coagulation bath (DI water in this study), which might produce the fully developed macrovoids at the bottom for membranes TS2–TS4. Moreover, the formation of SDS-TiO_2_ micelles decreased the interaction between polymer chains, diminishing the growth of the skin layer and improving the formation of macrovoids in the support layer [26]. The large macrovoids in membrane TS2 and the considerable closed macrovoids in membrane TS4 might impede the water permeability under pressure, which was in accordance with the result shown in Figure 4a. The highest cubical porosity of membrane TS3 shown in Figure 4b also benefited the highest permeability, which was increased by 8.6% compared to that of membrane TS1. Moreover, the overdosage SDS adsorbed on the membrane surface might block the channel, resulting in the decrease in porosity and permeability for membrane TS4 [27].

The roughness of membrane surfaces observed by AFM and mechanical strength characterized by elongation at break and tensile strength are shown in Figure 4c,d. Compared to the original PVDF/TiO_2_ membrane TS1, the membrane surface was modified smoothly by the addition of SDS. Adsorption of the negatively charged SDS on nanoparticle surfaces might result in a more uniform distribution on the membrane surface owing to the greater electrostatic repulsion between SDS-TiO_2_ micelles [28]. The lowest Ra, Rq and Rmax shown in Figure 4c for membrane TS3 illuminated the best surface roughness modification, which might result from the smoothing by the harmonious SDS-TiO_2_ micelle. The tensile strength of membranes TS1–TS4 (shown in Figure 4d) fluctuated around 31.5 MPa, manifesting no obvious difference. In terms of the mixed-matrix membrane preparation, the strong agglomeration of a nanosized addition often results in a decrease in mechanical properties [17]. PVDF/SDS-TiO_2_ composite membrane TS1 exhibited the weakest mechanical property of elongation at break. Compared to membrane TS1, the elongation at break was clearly enhanced by the addition of SDS for membranes TS2–TS4. The result might be attributed to the cross-linking of SDS in the micelles with PVDF polymers. With the increase in SDS, more cross-linking sites were provided, which probably benefited the improvement of membrane mechanical strength. The highest elongation at break for membrane TS3 might result from the closest cross-linking interaction between polymer matrix and harmonious SDS-TiO_2_ micelles. The overdosage of SDS in membrane TS4 might result in the sediment of SDS-TiO_2_ micelles in the matrix, which might lead to the very uneven cross-section structure and weak mechanical strength.

The results of the contact angle and Zeta potential of membranes TS1–TS4 are shown in Figure 4e,f. The hydrophilicity of membrane TS2–TS4 increased initially compared to membrane TS1 and decreased when the mass ratio of SDS/TiO_2_ exceeded 1 (see TS4 membrane). Contact angle of membrane TS3 was reduced by 13.3% with the modification of SDS-TiO_2_ micelles. Compared to membrane TS1, the negative Zeta potentials on the surface of membranes TS2 and TS3 increased slightly by the addition of the negatively charged SDS as an anionic surfactant. The negative Zeta potential on the surface of membranes TS3 was improved by 5.1% compared to the initial membrane TS1. The relatively low negative charge on membrane TS4 might be attributed to the sedimentation of SDS-TiO_2_ micelles. The most negative Zeta potential for membrane TS3 presumably caused by the evenly formed micelles, which might take full advantage of the evolving negatively charged SDS [29]. Hydrophobic interactions between the SDS molecules and the negatively charged membrane surface also further result in significant SDS adsorption and more negative membrane potential [30]. Compared with our previous modification by optimal PEG-TiO_2_ micelles, permeability and hydrophilicity of membrane TS3 was obviously enhanced, even though the porosity was relatively decreased [14].

### 3.3. Surface Composition of Membranes

**Figure 5:**
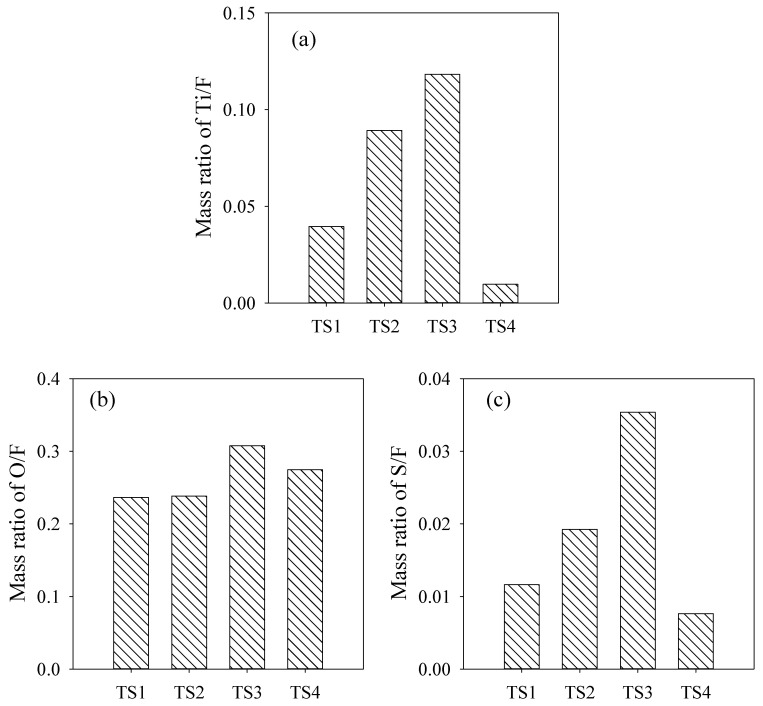
Mass ratio of Ti/F (**a**), O/F (**b**) and S/F (**c**) for membranes TS1–TS4 obtained by EDX determination, respectively.

EDX mapping was applied to examine the dispersion status of TiO_2_ and SDS-TiO_2_ micelles on membrane surfaces. The mass ratio of elements for membranes TS1–TS4 are shown in Table S1 and Figure 5. Ti/F, O/F and S/F were obtained to represent the distribution of TiO_2_, SDS/TiO_2_ and SDS on membrane surfaces, respectively. The highest Ti/F and O/F manifested the most SDS-TiO_2_ micelles dispersed on the surface of membrane TS3, illustrating the excellent hydrophilicity and negative Zeta potential. The minimum Ti/F and S/F demonstrated the least distribution of SDS and TiO_2_ on the membrane surface, which were supposed to verify the sedimentation of SDS-TiO_2_ micelles in the sub-layer or bottom layer of membrane TS4. XPS was also used to investigate the elemental compositions of membranes TS1–TS4, and the full spectra are shown in Figure S2. The uppermost peak for O1s (shown in Figure S3a) and S2p determined by XPS (shown in Figure S3c) further verified the preferred distribution of SDS-TiO_2_ micelles on the surface of membrane TS3, which might benefit the enhancement of hydrophilicity and negatively charged Zeta potential and thus the antifouling performance in filtration application. SDS-TiO_2_ micelles on the surface of membrane did not increase as the SDS dosage in the casting solution increased [11]. No obvious peak for Ti1s (shown in Figure S3b) might be owing to the low addition of TiO_2_ nanoparticles in all casting solutions.

### 3.4. Membrane Antifouling Performance

**Figure 6:**
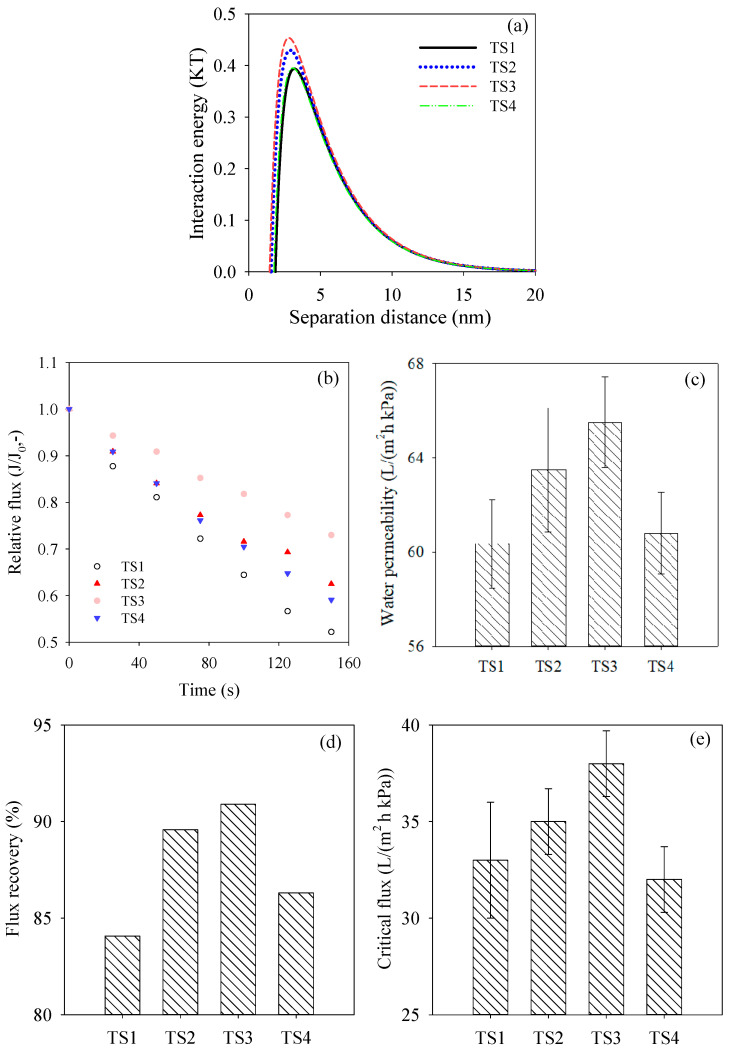
Membrane fouling behavior evaluating (*n* = 3): (**a**) interaction energy between membrane surfaces and approaching model foulants, (**b**) relative flux of membranes TSI–TS4 during filtrating BSA solution process, (**c**) water permeabilities of BSA fouled-membranes TS1–TS4, (**d**) flux recovery after washing process and (**e**) critical fluxes of membranes TS1–TS4 determined in MBR.

X-DLVO theory was employed to evaluate the antifouling performance of composite membranes TS1–TS4 in this study. BSA was used as a model foulant with an average size of 322.9 nm and Zeta potential of −10.3 eV (shown in Table S2). Contact angles of BSA and composite membranes TS1–TS4 determined by employing three probe liquids are displayed in Table S3. Table 2 shows the surface tension parameters of membranes and BSA, free energy of cohesion, as well as adhesion of membranes. The increasing electron donor components (γ^−^) might be attributed to the homogeneous adsorption of negatively charged SDS. The result for membrane TS3 might additionally result from the preferable dispersion of SDS-TiO_2_ micelles on the surface. The negative free energy of cohesion (∆G_121_^SWS^) and adhesion (∆G_123_^SWS^) per unit area of membranes were all negative, implying the attractive tendency of foulants on membrane surfaces [31]. The highest ∆G_121_^SWS^ and ∆G_123_^SWS^ values shown in membrane TS3 indicated the weakest attraction towards foulants, suggesting the best antifouling ability. As shown in Figure 6a, the highest interaction energy between membrane surface and approaching foulants further demonstrated the highest repulsive interaction towards foulants and superior antifouling performance for membrane TS3. The decrease in fouling potential at the appropriate mass ratio of SDS/TiO_2_ might be owing to the heterogeneities’ adsorption of negatively charged SDS on the surface of nanoparticles, which increases the repulsive interaction between membrane surface and negatively charged foulants. The high ∆G_121_^SWS^ and ∆G_123_^SWS^ values, as well as the interaction energy between membrane surface and approaching foulants obtained by X-DLVO theory, indicated the alleviative attachment of foulants on membrane surfaces during real filtration, promising an excellent antifouling trend. The smallest roughness might also benefit the enhancement of the antifouling performance, benefiting from the mitigation of the aggregation of foulants on the membrane surface [32]. The excellent antifouling performance for membrane TS3 was also proved by the lowest declining tendency during the filtration process of BSA solution (shown in Figure 6b). The highest permeability (see Figure 6c) and flux recovery (see Figure 6d) demonstrated the ascendant recovery capability after the fouling process. The relative flux for membrane TS3 was also evidently improved compared to the result for membranes modified with PEG-TiO_2_ micelles [14].

Critical flux measured in an actual MBR device is commonly recommended for reducing membrane fouling propensity [33]. In Figure 6e, it can be observed that with the increase in SDS, the critical flux was enhanced gradually. Critical flux of membrane TS3 was 38 L/(m^2^·h·kPa), which was increased by 15.2% compared to that of membrane TS1. Membrane TS3 showed the greatest antifouling ability, which was in accordance with the results evaluated by X-DLVO theory and foulants filtration experiment. Critical flux of membrane TS4 was obviously decreased compared to other membranes. The undesirable critical flux for membrane TS4 might be owing to the decreased hydrophilicity and negative Zeta potential. Low environmental risk was supposed to happen after filtration process in MBR application, owing to nontoxic property and small dosages of SDS and TiO_2_.

## 4. Conclusions

The various amount of anionic surfactant SDS was primely used as a dispersing agent of TiO_2_ nanoparticles to fabricate SDS-TiO_2_ micelles for the modification of antifouling performance of PVDF membranes. The low backscattering intensity and TSI monitored by multiple light scattering spectroscopy exhibited excellent stability of the composite casting solution with the mass ratio of SDS/TiO_2_ of 1:1. SDS-TiO_2_ micelles were supposed to mitigate the agglomeration of TiO_2_ nanoparticles evidently. Permeability, hydrophilicity and negative Zeta potential was enhanced by 8.6%, 13.3% and 5.1%, respectively. The successful immobilization of SDS-TiO_2_ micelles on the membrane surface was confirmed by the results of both EDX and XPS. The enhanced antifouling performance was verified by the high electron donor components (γ^−^), low negative free energy of surface cohesion and adhesion, high interaction energy between membrane surface and approaching foulants, and low decline of relative flux during the filtrating process of foulants. High critical flux determined in MBR also demonstrated the facile modification of antifouling ability by introducing the well-designed negatively charged SDS-TiO_2_ micelles in this study.

## Figures and Tables

**Table 1:** Detailed compositions of membranes TS1–TS4 (unit: wt.%).

Membranes	PVDF	DMSO	DMAc	PEG	TiO_2_	SDS
TS1	8	43	43	6	0.15	0
TS2	8	43	43	6	0.15	0.075
TS3	8	43	43	6	0.15	0.15
TS4	8	43	43	6	0.15	0.3

**Table 2:** Surface tension parameters and surface free energy of membranes at the separation distance of *h_0_* (0.157 ± 0.009 nm) (*n* = 3).

**Surface Tension Parameters for Each Membrane (mJ/m^2^)**							
Sample No.	*γ* ^LW^	*γ* ^+^	*γ^−^*	*γ* ^AB^		*γ* ^TOT^	
TS1	33.42 ± 0.21	0.52 ± 0.10	4.24 ± 0.55	2.96 ± 0.10		36.38 ± 0.29	
TS2	31.78 ± 0.03	0.22 ± 0.03	12.19 ± 0.97	3.28 ± 0.13		35.06 ± 0.13	
TS3	33.56 ± 0.06	0.09 ± 0.02	14.23 ± 1.75	1.81 ± 0.39		35.38 ± 0.43	
TS4	33.91 ± 0.18	0.08 ± 0.04	7.53 ± 0.77	1.50 ± 0.35		35.40 ± 0.17	
BSA	35.14 ± 1.13	0.28 ± 0.14	17.01 ± 1.87	3.81 ± 2.06		38.95 ± 1.41	
	**The Free Energy of Cohesion of** **Membranes (mJ/m^2^)**			**The Free Energy of Adhesion of Membranes (mJ/m^2^)**			
Membrane No.	Δ*G*_121_^LW^	Δ_121_*G*^AB^	Δ*G*_121_^SWS^	Δ*G*_123_^LW^	Δ*G*_123_^AB^		Δ*G*_123_^SWS^
TS1	−2.47 ± 0.08	−51.78 ± 1.49	−54.26 ± 1.53	−2.80 ± 0.05	−35.20 ± 1.07		−38.00 ± 1.09
TS2	−1.87 ± 0.01	−28.57 ± 2.42	−30.45 ± 2.43	−2.44 ± 0.01	−22.68 ± 1.22		−25.12 ± 1.23
TS3	−2.89 ± 0.03	−22.37 ± 4.13	−25.26 ± 4.16	−3.03 ± 0.01	−19.49 ± 1.92		−22.51 ± 1.94
TS4	−2.66 ± 0.07	−44.06 ± 1.98	−46.73 ± 1.91	−2.91 ± 0.04	−29.83 ± 1.12		−32.74 ± 1.08

## Data Availability

The original contributions presented in this study are included in the article and Supplementary Material. Further inquiries can be directed to the corresponding authors.

## Supplementary Materials

The following supporting information can be downloaded at: https://www.mdpi.com/article/10.3390/membranes15110330/s1, Figure S1: Surface porosity (a) and pore size distribution (b–e) of membranes TS1–TS4; Figure S2: Binding energy of membranes TS1–TS4 determined by XPS; Figure S3: Peaks of O1s (a), Ti1s (b) and S2p (c) on the surfaces of membranes TS1–TS4 determined by XPS; Table S1: Physicochemical characteristics of the influent wastewater for critical flux measurement; Table S2: Mass ratio of elements for membranes TS1–TS4 determined by EDX; Table S3: Properties of BSA (*n* = 3); Table S4: Contact angle of membranes and BSA determined by employing three probe liquids (*n* = 3).
