# Supplementary material for: Fouling Mitigation of PVDF Membrane Induced by Sodium Dodecyl Sulfate (SDS)-TiO2 Micelles
Source: Membranes (Basel). 2025 Oct 30;15(11):330. doi: 10.3390/membranes15110330 (PMC12654714; doi:10.3390/membranes15110330)
Supplement: Supplementary file 1 [file membranes-15-00330-s001.zip › membranes-3896859-supplementary.docx]

Fig. S1 Surface porosity (a) and pore size distribution (b-e) of membranes TS1-TS4.

Table S1 Physicochemical characteristics of the influent wastewater for critical flux measurement.

| Item | COD (mg/L) | Ammonium (mg/L) | TN (mg/L) | TP (mg/L) | pH |
| --- | --- | --- | --- | --- | --- |
| Influent wastewater | 381.1±8.4 | 32.0±2.4 | 32.4±2.9 | 5.5±0.5 | 6.7±0.2 |

Table S2 Mass ratio of elements for membranes TS1-TS4 determined by EDX.

| Element | TS1 | TS2 | TS3 | TS4 |
| --- | --- | --- | --- | --- |
| C | 44.72 | 38.66 | 38.31 | 51.57 |
| N | 11.01 | 10.19 | 14.61 | 9.53 |
| O | 8.12 | 9.04 | 9.91 | 8.26 |
| F | 34.39 | 37.99 | 32.22 | 30.11 |
| S | 0.4 | 0.73 | 1.14 | 0.23 |
| Ti | 1.36 | 3.39 | 3.81 | 0.29 |

Fig. S2 Binding energy of membranes TS1-TS4 determined by XPS.

Fig. S3 Peaks of O1s (a), Ti1s (b) and S2p (c) on the surfaces of membranes TS1-TS4 determined by XPS.

Table S3 Properties of BSA (*n*=3).

|  | Concentration (g/L) | Average size (nm) | Zeta potential (mV) |
| --- | --- | --- | --- |
| BSA | 1.0 | 322.9±4.4 | -10.3±0.3 |

Table S4 Contact angle of membranes and BSA determined by employing three probe liquids (*n*=3).

| Membrane | Water | Formamide | Diiodomethane |
| --- | --- | --- | --- |
| TS1 | 82.7±0.5 | 59.5±0.7 | 51.5±0.4 |
| TS2 | 74.2±1.0 | 60.1±0.3 | 54.4±0.1 |
| TS3 | 71.7±1.4 | 60.1±0.3 | 49.7±0.1 |
| TS4 | 80.5±0.6 | 62.8±0.4 | 50.7±0.3 |
| BSA | 66.2±2.4 | 52.7±1.8 | 48.4±2.0 |
